# Exploratory Associations Between Handgrip Strength, Fatigue State, and Cycling Performance in Trained Cyclists

**DOI:** 10.3390/sports14100423

**Published:** 2026-10-01

**Authors:** Daniel Gotti, Roberto Codella, Matteo Moraschini, Andrea Meloni, Antonio La Torre, Luca Filipas

**Affiliations:** 1 Department of Biomedical Sciences for Health, Università Degli Studi di Milano, 20133 Milan, Italy; daniel.gotti@unimi.it (D.G.);; 2 Department of Endocrinology, Nutrition and Metabolic Diseases, IRCCS MultiMedica, 20138 Milan, Italy; 3 TotalEneergies Pro Cycling Team, 85140 Essarts-en-Bocage, France

**Keywords:** handgrip strength (HGS), Hooper Index, endurance performance, fatigue and recovery, high-intensity and long-duration exercise

## Abstract

Practical readiness monitoring requires rapid, low-cost, and accessible measures. This exploratory repeated-measures study investigated whether the handgrip strength test (HGS) reflects short-term changes in fatigue and readiness in trained cyclists. Twenty-two trained male cyclists completed laboratory testing after rest, easy training, high-intensity training, and long-duration exercise. HGS at 90° and 180° elbow flexion, Hooper Index (HI), heart rate variability (HRV), physiological responses, blood lactate kinetics, power decrement, and cycling performance were assessed. Cycling performance and exercise heart-rate responses differed across conditions (*p* < 0.05). HGS_90_ also differed across conditions (*p* = 0.030), whereas HGS_180_ did not (*p* = 0.133). Higher HGS was associated with greater short-duration cycling power, particularly the 30 s performance, even after adjustment for body mass. However, within/between-athlete mixed models showed that these relationships primarily reflected stable differences between athletes: day-to-day HGS fluctuations were not associated with corresponding changes in performance, peak blood lactate, HRV, lactate clearance, or power decrement. Furthermore, HGS did not provide significant incremental information beyond condition, body mass, HI, and HRV. HGS may therefore complement assessments of cyclists’ power profiles, but the present findings do not support its use as an independent marker of acute readiness.

## 1. Introduction

Road cycling imposes high and variable training demands, particularly in highly trained and elite populations [1], requiring coaches to balance overload, recovery, and competition readiness [2]. Consequently, precise and systematic training programming is fundamental to optimize performance and manage physiological stress [2], particularly the capacity to assign high-intensity sessions—which mostly drive performance improvements [3]—exclusively on days when the athlete is physiologically ready to tolerate them.

Readiness and fatigue concepts are related but not synonymous constructs. Readiness refers to the athlete’s current capacity to perform a planned task, whereas fatigue is one of several factors that may reduce that capacity [4].

Common readiness monitoring approaches include performance-based tests such as countermovement jump (CMJ) or short sprints, which are widely used in team sports [5]; perceptual readiness questionnaires such as the Hooper Index [6,7], autonomic markers such as heart rate variability (HRV)—easily used in endurance sports, which provide information regarding cardiac parasympathetic modulation and its day-to-day variation in response to training and recovery—[8], and biochemical measures such as testosterone, cortisol or creatine kinase analysis [9]. Despite this broad landscape, no single measure provides a comprehensive assessment of athlete readiness, as each approach captures a different component of the recovery–fatigue continuum [5].

The handgrip strength test (HGS) is an affordable, portable, rapid-to-administer test, and can be measured reliably when posture, handle position, and instructions are standardized [10,11,12,13]. Although HGS is not a cycling-specific performance determinant, it reflects general strength and neuromuscular function and may therefore be considered a candidate complementary readiness measure [14,15]. Evidence for its sensitivity to acute fatigue remains limited and inconsistent; notably, Jaen-Carrillo et al. reported no change in the HGS after an elite trail-running competition [16]. These negative findings warrant further investigation under controlled repeated-measures conditions to determine whether HGS is genuinely unrelated to acute changes in fatigue and performance, or whether potentially meaningful associations may emerge when within-athlete variability and relevant confounding factors are appropriately considered.

The overall aim of this exploratory study was to investigate whether HGS is sensitive to short-term variations in the recovery–fatigue status in trained cyclists and whether it is associated with subsequent cycling performance. Specifically, the primary aims were to evaluate changes in standardized HGS following training stimuli differing in volume and intensity (see condition in Section 2) and to examine its association with cycling performance, particularly short-duration maximal power and 5 min performance. Secondary exploratory aims were to determine whether within-athlete changes in the HGS tracked corresponding changes in performance, whether the HGS–performance relationship differed across training conditions, and whether the HGS provided additional information beyond body mass and established readiness measures such as HI and HRV. Additional exploratory analyses examined associations between HGS and autonomic, perceptual, heart-rate, and lactate-related outcomes.

## 2. Materials and Methods

### 2.1. Participants

Participant inclusion criteria were as follows: (1) self-reported RPO_20_ > 3.5 W·kg^−1^; (2) training volume > 7 h·week^−1^ and at least 4 sessions per week; (3) cycling experience > 3 years; and (4) absence of any known disease or exercise limitations during the previous 3 months. The study protocol was approved by the Research Ethics Committee of the University of Milan (approval number 52/20, attachment 4) and conducted in accordance with the ethical standards of the Declaration of Helsinki. Twenty-five trained [17] male cyclists were recruited for the study. Subsequently, two participants were excluded due to non-compliance with the prescribed training sessions, and one participant withdrew from the study due to the onset of health issues, resulting in a final sample of 22 participants (36.2 ± 9.6 years; 182.4 ± 7.7 cm; 73.7 ± 8.5 kg; 11.3 ± 3.1 h·week^−1^ of training; 4.5 ± 0.7 W·kg^−1^ self-reported power over 20 min—RPO_20_). All participants received written information regarding the study procedures and potential risks and provided written informed consent prior to participation.

### 2.2. Sample Size Estimation and Justification

An a priori sample-size calculation was conducted for the repeated-measures component of the study, corresponding to the primary aim of evaluating changes in standardized HGS and cycling-performance outcomes across the four experimental conditions. The calculation was performed using G*Power (version 3.1.9.7), assuming α = 0.05, statistical power = 0.85, a moderate effect size (Cohen’s f = 0.25), a correlation among repeated measures of 0.60, and a nonsphericity correction (ε) of 1.0. Under these assumptions, a minimum sample of 20 participants was required. Cohen’s f = 0.25 was selected as a conventional moderate effect size for this planning calculation [18]. The final analytical sample comprised 22 participants and therefore exceeded the minimum sample size required for the repeated-measures component.

The second primary aim, concerning the association between the HGS and subsequent cycling performance, was not separately powered at the design stage. Accordingly, these association analyses were interpreted with particular emphasis on effect estimates and 95% confidence intervals. The within-athlete analyses, HGS × condition interaction models, and models evaluating the incremental information provided by the HGS beyond body mass, HI, and HRV were considered secondary exploratory analyses.

### 2.3. Study Design and Familiarization

Once the participants were selected, a six-week randomized repeated-measures study was conducted following the temporal schedule illustrated in Figure 1. During the first two weeks, participants underwent a familiarization period in which they completed the entire testing procedure twice, becoming accustomed to the HGS (adjusting the grip width according to personal comfort) and to the evaluation session. Throughout this introductory phase, participants were instructed in the use of the Elite HRV application [19] and in completing the Hooper Index questionnaire [7]. A total of 2 weeks of baseline data were collected prior to the experimental phase to provide reference values for the experimental data and to fully familiarize participants with the morning assessment routine. After this period, participants completed all four experimental conditions in a counterbalanced manner, with at least 72 h between laboratory sessions to reduce potential carry-over effects [20]. A comprehensive overview of the experimental conditions is presented in Table 1 and in Section 2.4. Condition order was randomized using a computer-generated random allocation sequence. Each condition was completed once; therefore, within-condition reproducibility could not be quantified.

### 2.4. Experimental Conditions and Test Procedures

During the experimental month, participants were tested weekly. Each laboratory session was preceded by two days of imposed training. At 48 h before testing, participants complete a standardized 90 min easy ride. At 24 h prior testing, participants complete one of four specific conditions: (1) a complete rest day (RD), (2) an easy training day—active recovery with a rate of perceived exertion (RPE) on the Borg CR10 RPE [21] < 3 (ED), (3) a high-intensity training (HIT) session consisting of 5 × 5 min at maximal power (HD), and (4) a long-duration session at least as long as the participant’s usual weekly long ride (LD).

All prescribed training sessions were performed outdoors during the morning on the participants’ own bicycles. On non-prescribed training days, participants were allowed to follow their habitual training routines but were instructed to avoid sessions exceeding the duration of the long-duration condition or the intensity of the high-intensity condition. Daily training and self-reported readiness were monitored throughout the experimental period to identify substantial deviations from the study protocol and to minimize the potential influence of additional training load on the experimental sessions. On each testing day, the following procedures were carried out. Upon waking, on a 5 min period, participants measured heart rate variability (HRVmorning), resting heart rate (HRmorning), and completed the Hooper Index (HI) questionnaire—assessing sleep quality and the quantities of stress, fatigue and muscle soreness on a 1–7 likert scale [7], submitting all data via an online form. Notably, this morning routine was carried out every day. The HI was calculated as the sum of the scores across its four dimensions. Participants were instructed to maintain a consistent breakfast routine and avoid caffeine/stimulants on testing [22] and laboratory testing was scheduled within a ±2 h window for each participant [23]. In the laboratory, body mass was first recorded. Three maximal HGS trials per arm were performed with the elbow flexed at 90° (HGS_90_) and with the arm fully extended at 180° (HGS_180_) [14], each lasting 5 s with 30 s recovery. The trials were randomized between position and verbal encouragement was provided. Following the HGS assessment, participants performed the cycling protocol on their own bicycles mounted on a smart trainer (see materials section). After a standardized 10 min warm-up at 100 w—including three short self-paced accelerations—followed by 2 min of easy pedaling, participants completed a seated 30 s all-out test, preceded by a 30 s rolling phase at 4.5 W·kg [24]. A 15 min recovery phase at 100 W followed, during which capillary blood lactate was sampled every two minutes to characterize peak, time-to-peak, and clearance. Participants then performed a seated 5 min all-out test, with heart rate and power output hidden from view, as a surrogate MAP [25]. Heart rate recovery (HRR) was recorded at 1 and 3 min post-exercise, following the formula [26]:

HRR = HRPeak − HR_(1/2/3 min)

Finally, RPE was recorded at the end of the entire session using the Borg CR10 scale [27]. The full testing and training schedule is illustrated in Figure 2.

### 2.5. Materials

HRmorning and HRVmorning data were collected using the Elite HRV mobile application (Elite HRV, Asheville, NC, USA) connected to a Garmin chest strap HRM-Dual, Garmin Ltd., Olathe, KS, USA. Data submission was performed through Google forms, which were also used for the administration of the Hooper Index questionnaire. HGS was assessed using a digital hand dynamometer (Zhongshan Camry Electronic Co., Ltd., Zhongshan, Guangdong, China). Each participant used their own bicycle, adjusted according to their habitual setup, and mounted on a smart trainer (Direto XR-T, Elite, Fontaniva, Italy) connected to My E-Training APP (Direto XR-T, Elite, Fontaniva, Italy). Heart rate during cycling tests was continuously recorded using a chest strap monitor (HRM-Dual, Garmin Ltd., Olathe, KS, USA), with battery levels checked before each session. Blood lactate concentration was measured using a portable lactate analyzer (Lactate Pro2, Arkray KDK, Kyoto, Japan).

### 2.6. Data Processing and Outcome Definitions

For each condition, the following variables were collected: body mass, HRVmorning, HRmorning, HI, HGS_90_, HGS_180_, maximal mean power over 5 s (MMP_5s_) and 30 s (MMP_30s_), the 30 s power decrement index (PDI), maximal mean power over 5 min (MMP_5min_), blood lactate peak (BLaPeak), time to blood lactate peak (TTP-BLa), blood lactate clearance (BLaClearance), RPE, time-trial heart rate peak (TT HRpeak), mean time-trial heart rate (TT HRmean), time-trial cadence (TT RPM), and HRR_1min_ and HRR_3min_. BLaPeak was defined as the post-sprint interpolated point with highest lactate concentration, TTP-BLa as the elapsed time to reach this peak, and BLaClearance as the amount of lactate washed out per min. HI was interpreted such that higher values reflected a more favorable wellness/readiness state. HRR was calculated as peak exercise heart rate minus heart rate measured during recovery (at 1 and 3 min). The PDI was calculated for each condition as:

PDI = [(maximum 5-s power − minimum 5-s power)/maximum 5-s power] × 100

with higher values indicating a greater decline during the 30 s all-out test. Compliance with the prescribed training conditions was verified using training files recorded by the participants’ cycling computers. Each session was individually reviewed using TrainingPeaks and WKO to confirm adherence to the prescribed training protocol.

### 2.7. Statistical Analysis

Data are presented as mean ± standard deviation (SD). One-way repeated-measures ANOVA compared RD, ED, HD, and LD, sphericity was tested and Greenhouse–Geisser correction applied when violated. Post hoc pairwise comparisons used a single Sidak-adjusted procedure; paired Cohen’s dz was reported for significant contrasts (approximately 0.2 small, 0.5 medium, and 0.8 large). Easy-condition associations between HGS and cycling power were evaluated using Pearson correlations, Spearman sensitivity analyses, partial Pearson correlations controlling for body mass, and relative HGS/kg versus power/kg sensitivity analyses. To distinguish stable differences between cyclists from day-to-day changes, random-intercept mixed models decomposed HGS and readiness predictors into between-athlete means and within-athlete deviations; condition and body mass were included where appropriate. HGS-within × condition interactions tested whether the HGS–performance slope differed across conditions. Exploratory condition-adjusted mixed models evaluated three predictors (HGS_90_, HGS_180_ and HI) across 14 outcomes and were corrected using the Benjamini–Hochberg false-discovery rate across 42 tests. Finally, nested multivariable mixed models tested whether adding HGS_90_ or HGS_180_ improved MMP_5s_, MMP_30s_, or MMP_5min_ beyond condition, body mass, HI, and HRV using likelihood-ratio tests and AIC. Statistical significance was set at *p* < 0.05 (two-tailed), with exploratory inferences interpreted alongside 95% confidence intervals, effect sizes, and FDR-adjusted q-values. All analyses were performed using SPSS (IBM SPSS Statistics, Armonk, NY, USA, version 29.0.2.0).

## 3. Results

### 3.1. HGS Responses Across Experimental Conditions

Standardized HGS_90_ differed across conditions (F(3,48) = 3.23, *p* = 0.030, partial eta^2^ = 0.168), with ED higher than RD after Sidak adjustment (56.84 ± 9.62 vs. 55.11 ± 9.09 kg, adjusted *p* = 0.008), HGS_90_ was also numerically lower in HD than in ED (54.97 vs. 56.84 kg; −3.3%), although this pairwise difference did not remain significant after Sidak adjustment (adjusted *p* = 0.187). HGS_180_ did not show a significant condition effect (F(3,48) = 1.96, *p* = 0.133, partial eta^2^ = 0.109). Descriptive values and omnibus effects for all outcomes are summarized in Table 2.

### 3.2. Cycling Performance and Readiness

Cycling performance showed condition effects for MMP_5s_ (F(3,48) = 3.17, *p* = 0.033, partial eta^2^ = 0.165), MMP_30s_ (F(3,48) = 4.92, *p* = 0.0046, partial eta^2^ = 0.235), and MMP_5min_ (F(3,48) = 9.83, *p* < 0.001, partial eta^2^ = 0.381). No pairwise MMP_5s_ contrast survived adjustment. MMP_30s_ was higher in RD and ED than HD (adjusted *p* = 0.016 and 0.044), and MMP_5min_ was higher in RD and ED than HD (*p* = 0.0020 and 0.0018).

Peak and mean heart rate during the 5 min test also differed across conditions (HRpeak: F(3,48) = 10.60, *p* < 0.001, partial eta^2^ = 0.398; HRmean: F(3,48) = 11.14, *p* < 0.001, partial eta^2^ = 0.410), with lower responses after HD/LD relative to RD/ED in the significant contrasts. No significant condition effects were found for BLaPeak (*p* = 0.176), TTP-BLa (*p* = 0.608), BLaClearance (*p* = 0.764), PDI (F(3,48) = 0.035, *p* = 0.991, partial eta^2^ = 0.002), RPE (*p* = 0.875), cadence (*p* = 0.191), or HRR1 (*p* = 0.298). HRR3 approached but did not reach significance (*p* = 0.060). Mean PDI values were 38.25 ± 8.51%, 38.66 ± 7.91%, 38.40 ± 8.28%, and 38.46 ± 8.44% for RD, ED, HD, and LD, respectively.

HI showed a large condition effect (F(3,48) = 6.95, *p* < 0.001, partial eta^2^ = 0.303), with RD higher than HD (adjusted *p* = 0.019) and LD (*p* = 0.012). HRVmorning was the only outcome violating the sphericity assumption (Mauchly’s *p* = 0.014; ε = 0.60) and did not differ across conditions after Greenhouse–Geisser correction (F(1.80,28.77) = 1.47, *p* = 0.247, partial eta^2^ = 0.084), and morning resting heart rate was also unchanged (*p* = 0.459).

The main between-condition comparisons are presented in Figure 3.

### 3.3. HGS Associations with Cycling Performance

In ED, absolute HGS was strongly associated with short-duration cycling power. HGS_90_ correlated with MMP_5s_ (r = 0.666, *p* = 0.004) and MMP_30s_ (r = 0.715, *p* = 0.001), whereas the association with MMP_5min_ was weaker and non-significant (r = 0.409, *p* = 0.103). HGS_180_ showed the same duration-dependent pattern. Because HGS was also associated with body mass, partial correlations were calculated. After controlling for body mass, HGS_90_ remained associated with MMP_30s_ (partial r = 0.534, *p* = 0.027) but not MMP_5s_ (partial r = 0.446, *p* = 0.073) or MMP_5min_ (partial r = 0.101, *p* = 0.699). HGS_180_ remained associated with MMP_5s_ (partial r = 0.493, *p* = 0.044) and MMP_30s_ (partial r = 0.661, *p* = 0.004), whereas its association with MMP_5min_ was non-significant (partial r = 0.273, *p* = 0.289). Relative HGS/kg versus MMP/kg sensitivity analyses produced the same general pattern, with the clearest association for 30 s performance, as shown in Figure 4.

Mixed models separating between-athlete HGS from within-athlete day-to-day deviations showed no significant within-athlete association between HGS_90_ and MMP_5s_ (*p* = 0.702), MMP_30s_ (*p* = 0.693), or MMP_5min_ (*p* = 0.924). The corresponding between-athlete HGS_90_ component was associated with MMP_5s_ (β = 6.29 W per kg HGS, *p* = 0.029) and MMP_30s_ (β = 5.37 W per kg HGS, *p* = 0.010), but not MMP_5min_ (*p* = 0.589). HGS_180_ showed the same qualitative distinction: between-athlete associations with sprint power were present, whereas within-athlete day-to-day effects were not. HGS-within × condition interactions were non-significant for MMP_5s_, MMP_30s_, and MMP_5min_ for HGS_90_ (all *p* ≥ 0.496) and HGS_180_ (all *p* ≥ 0.509), providing no evidence that the fatigue condition altered the HGS–performance slope. The PDI was not robustly associated with the HGS. In condition-adjusted pooled models, the associations were non-significant for HGS_90_ (β = 0.233 percentage points per kg, *p* = 0.104) and borderline for HGS_180_ (β = 0.298, *p* = 0.053); neither survived FDR correction. Within-athlete HGS deviations were unrelated to PDI (HGS_90_: *p* = 0.867; HGS_180_: *p* = 0.291). A nominal between-athlete association was observed for HGS_90_ (β = 0.370, *p* = 0.044), but it did not survive multiplicity correction and was attenuated after adjustment for body mass (*p* = 0.141).

Nested multivariable mixed models included condition and body mass, then added HI and HRVmorning, followed by HGS. Adding HGS_90_ did not improve model fit for MMP_5s_ (likelihood-ratio *p* = 0.349; ΔAIC = +1.90), MMP_30s_ (*p* = 0.268; ΔAIC = +1.37), or MMP_5min_ (*p* = 0.929; ΔAIC = +3.85). Sensitivity analyses were consistent for HGS_180_ (*p* = 0.384, 0.170, and 0.553, respectively). In the HGS_90_ full models, body mass was the only continuous covariate independently associated with all three performance outcomes (*p* = 0.013, 0.025, and 0.015). Adding HRVmorning jointly improved the MMP_30s_ model before HGS was entered (likelihood-ratio *p* = 0.026; ΔAIC = −3.31), although neither HRVmorning component was individually significant in the final model. These analyses provide no evidence that HGS adds independent day-to-day performance information beyond condition, body size, HI, and HRVmorning in this sample.

### 3.4. HGS Associations with Readiness and Physiological Outcomes

Within-athlete HI was not associated with HGS_90_ (*p* = 0.291) or HGS_180_ (*p* = 0.593), whereas within-athlete HI was associated with HRV (β = 0.830, *p* = 0.006) and showed a borderline inverse association with RPE (β = −0.107, *p* = 0.049). Across the 42 condition-adjusted exploratory tests, FDR-significant pooled associations were observed for HGS_90_ with BLaPeak (β = 0.104 mmol·L^−1^ per kg, *p* = 0.002, q = 0.039), HGS_180_ with BLaPeak (β = 0.111, *p* = 0.002, q = 0.039), and HGS_90_ with HRVmorning (β = 0.550 HRV units per kg, *p* = 0.003, q = 0.047). The pooled HGS_180_–HRV association was significant before multiplicity correction (β = 0.545, *p* = 0.008) but not after FDR correction (q = 0.083). Within/between decomposition showed significant between-athlete associations with BLaPeak for HGS_90_ (*p* = 0.003) and HGS_180_ (*p* = 0.005), but no corresponding within-athlete associations (*p* = 0.200 and *p* = 0.160). Similarly, HRV was associated with the between-athlete components of HGS_90_ (*p* < 0.001) and HGS_180_ (*p* = 0.003), but not with their within-athlete components (*p* = 0.714 and *p* = 0.695). No other significant association were found.

## 4. Discussion

### 4.1. Principal Findings

This exploratory repeated-measures study examined whether the HGS changes across preceding-day training conditions and whether the HGS provides information about subsequent cycling performance. Four findings are central.

First, standardized HGS_90_ showed a significant overall condition effect. However, the pairwise pattern did not support a straightforward reduction in HGS following the fatiguing conditions. HGS_90_ was numerically lower after HIT than after the easy condition (54.97 vs. 56.84 kg; −3.3%), but this difference did not remain significant after Sidak adjustment (adjusted *p* = 0.187). Similarly, HGS_90_ after the long-duration condition was numerically lower than after the easy condition (54.51 vs. 56.84 kg; −4.1%), although this comparison was also not significant after adjustment. The only significant pairwise difference was the higher HGS_90_ observed after the easy condition compared with the rest condition (adjusted *p* = 0.008). This pattern may indicate that HGS_90_ is influenced by the preceding training condition, but not through a simple fatigue-related suppression.

Second, absolute HGS was strongly associated with short-duration power across athletes, and the HGS-MMP_30s_ association persisted after body-mass adjustment.

Third, within-athlete day-to-day HGS changes did not track within-athlete changes in cycling performance, and fatigue condition did not modify the HGS–performance slope.

Fourth, the HGS did not add significant incremental information about performance after condition, body mass, HI, and HRV were considered simultaneously.

In addiction, the experimental conditions produced clear next-day performance differences. MMP_30s_ and MMP_5min_ were lower after HD than after RD/ED, and peak and mean heart-rate responses during the 5 min test were also lower after HD and/or LD. HI differentiated RD from HD and LD, whereas HRV and morning heart rate did not show significant condition effects. Nevertheless, the significant within-athlete association between HI and HRV (β = 0.830, *p* = 0.006) suggests that day-to-day changes in perceived readiness covaried with changes in cardiac autonomic modulation. This partial convergence does not indicate that the measures are interchangeable, as subjective and objective monitoring indices often show weak or inconsistent correspondence and HRV responses require longitudinal, athlete-specific interpretation [28,29]. Rather, the present findings support the combined use of perceptual and autonomic measures as complementary components of a multimodal monitoring framework.

### 4.2. HGS and Cycling Performance

The relationship between HGS and cycling performance appeared to be largely specific to short-duration efforts. Cyclists with greater HGS generally expressed higher 5 s and, particularly, 30 s power, whereas this relationship became progressively weaker when performance was extended to 5 min. Although body mass contributed to these associations, it did not fully explain the relationship with 30 s power, which remained evident after adjustment for body size. In contrast, the association with 5 min performance disappeared after accounting for body mass. Taken together, these findings suggest that the HGS may primarily reflect broader neuromuscular characteristics associated with strength and short-duration power production, rather than the physiological qualities underpinning more prolonged aerobic performance. This interpretation is consistent with the strong associations of HGS with lower-limb strength, countermovement-jump power, and muscle mass reported by McBride et al. [30], and with our finding that HGS was more strongly associated with 30 s cycling power than with 5 min performance. Conversely, although Ajepe et al. [31] observed a moderate unadjusted association between HGS and estimated $\overset{\cdot }{V}O_{2}$max, the HGS was not an independent predictor after accounting for sex, body mass index, and physical activity, further suggesting that its relationship with aerobic capacity may largely reflect broader between-individual characteristics rather than aerobic fitness.

### 4.3. Day-to-Day Readiness, Autonomic Measures, and Lactate

When considering day-to-day fluctuations in HGS, in both standardized positions (HGS_90_ and HGS_180_), and their relationship with performance, readiness, and other physiological responses, it is important to interpret the LMM findings alongside their decomposition into within- and between-athlete components. This distinction provides a different perspective on the associations observed in the pooled models. Cyclists with higher average HGS tended to produce greater sprint power and also showed higher BLaPeak and morning HRV. However, these relationships were not reproduced within individuals: when an athlete’s HGS was higher than his own usual level, this was not accompanied by better performance or corresponding changes in BLaPeak or HRV. Thus, the associations observed in the pooled models appear to reflect primarily stable differences between athletes rather than an acute day-to-day readiness signal. A similar picture emerged for the PDI, which did not differ across conditions and showed no consistent relationship with either HGS_90_ or HGS_180_; moreover, the isolated between-athlete association observed for HGS_90_ was weakened after accounting for body mass. Overall, these findings suggest that HGS may capture broader individual neuromuscular characteristics but provide limited support for interpreting its daily fluctuations as a direct marker of acute glycolytic readiness or sprint fatigability.

Finally, the inverse within-athlete association between HI and RPE (β = −0.107, *p* = 0.049) indicates that more favorable perceived-readiness scores were associated with lower perceived exertion during subsequent exercise. This finding is consistent with Haddad et al. [32], who showed that perceptual indicators included in the HI—particularly fatigue, stress, muscle soreness, and sleep quality—can influence RPE during standardized exercise, supporting the practical relevance of subjective wellness monitoring for interpreting athletes’ responses to training.

### 4.4. Limitations and Practical Applications

The absence of repeated exposures to each condition prevents assessment of within-condition reproducibility and determination of whether individual HGS changes are sufficiently reliable for daily decision-making. Moreover, HGS reflects general neuromuscular strength–power characteristics but does not directly assess the lower-limb musculature responsible for cycling performance. Its interpretation may also be confounded by local hand and forearm fatigue caused by prolonged braking, technical terrain, or road vibration. Practitioners should therefore standardize testing posture, grip setting, instructions, and time of day; interpret HGS relative to an athlete’s individual baseline; consider the upper-limb demands of the preceding session; and use HGS alongside perceptual, autonomic, and cycling-specific measures rather than as a standalone go/no-go criterion. Future studies should repeat each condition, establish reliability and meaningful-change thresholds, and compare HGS responses across road, gravel, and mountain-bike demands.

## 5. Conclusions

In trained cyclists, standardized HGS—particularly HGS_90_—showed some sensitivity to the preceding-day training condition and was associated cross-sectionally with short-duration cycling power, especially MMP_30s_. Although part of this sprint-related association remained after adjustment for body mass, the HGS was not consistently associated with 5 min power, lactate responses, or heart rate recovery. Moreover, day-to-day fluctuations in the HGS within the same athlete did not correspond with changes in cycling performance and provided no significant additional information beyond training condition, body mass, HI, and HRV. Collectively, these findings support HGS as a rapid, low-cost complementary measure of an athlete’s neuromuscular strength–power profile, but not yet as an independent marker of acute cycling readiness or performance. Its interpretation should also account for the specific upper-limb demands of the preceding cycling session. Prospective longitudinal studies across different cycling disciplines and training demands are therefore required before the HGS can be used to guide daily training decisions.

## Figures and Tables

**Figure 1:**
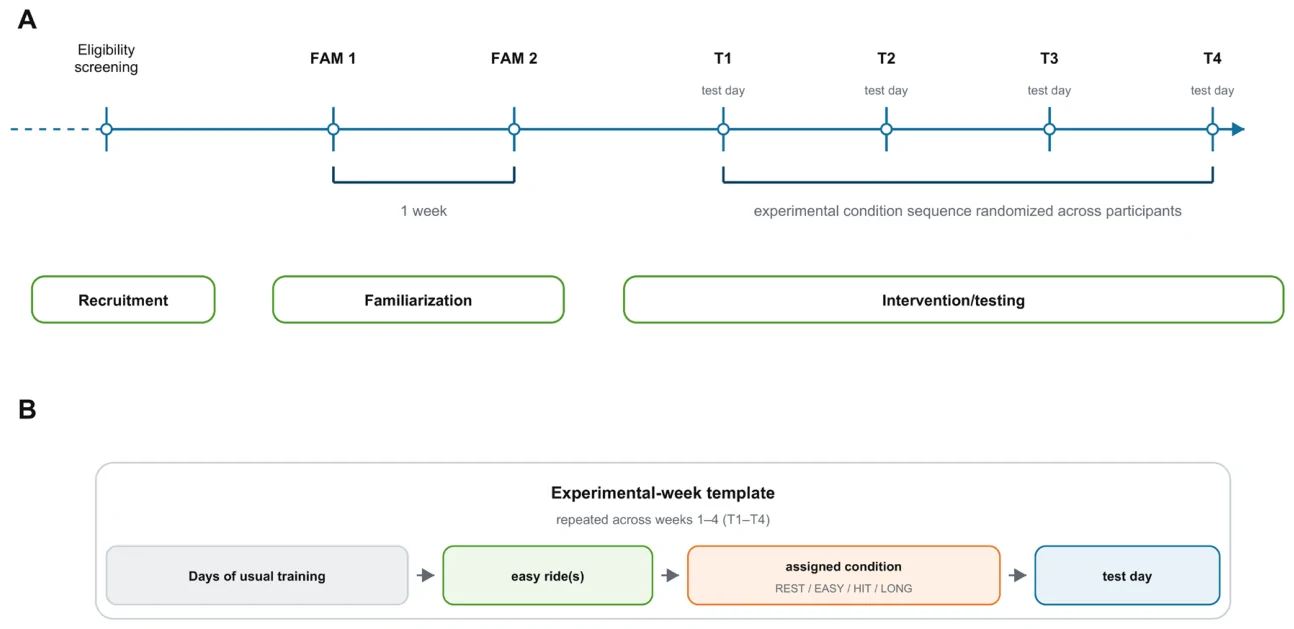
(**A**) Schematic representation of the study timeline. “Fam” = familiarization session. (**B**) Schematic representation of the study test-week; “Rest” = rest day; “Easy” = 90 min low-intensity ride; “HIT” = five 5 min intervals performed at maximal average power; “Long” = training session at least as long as the athlete’s usual weekly long-duration ride.

**Figure 2:**
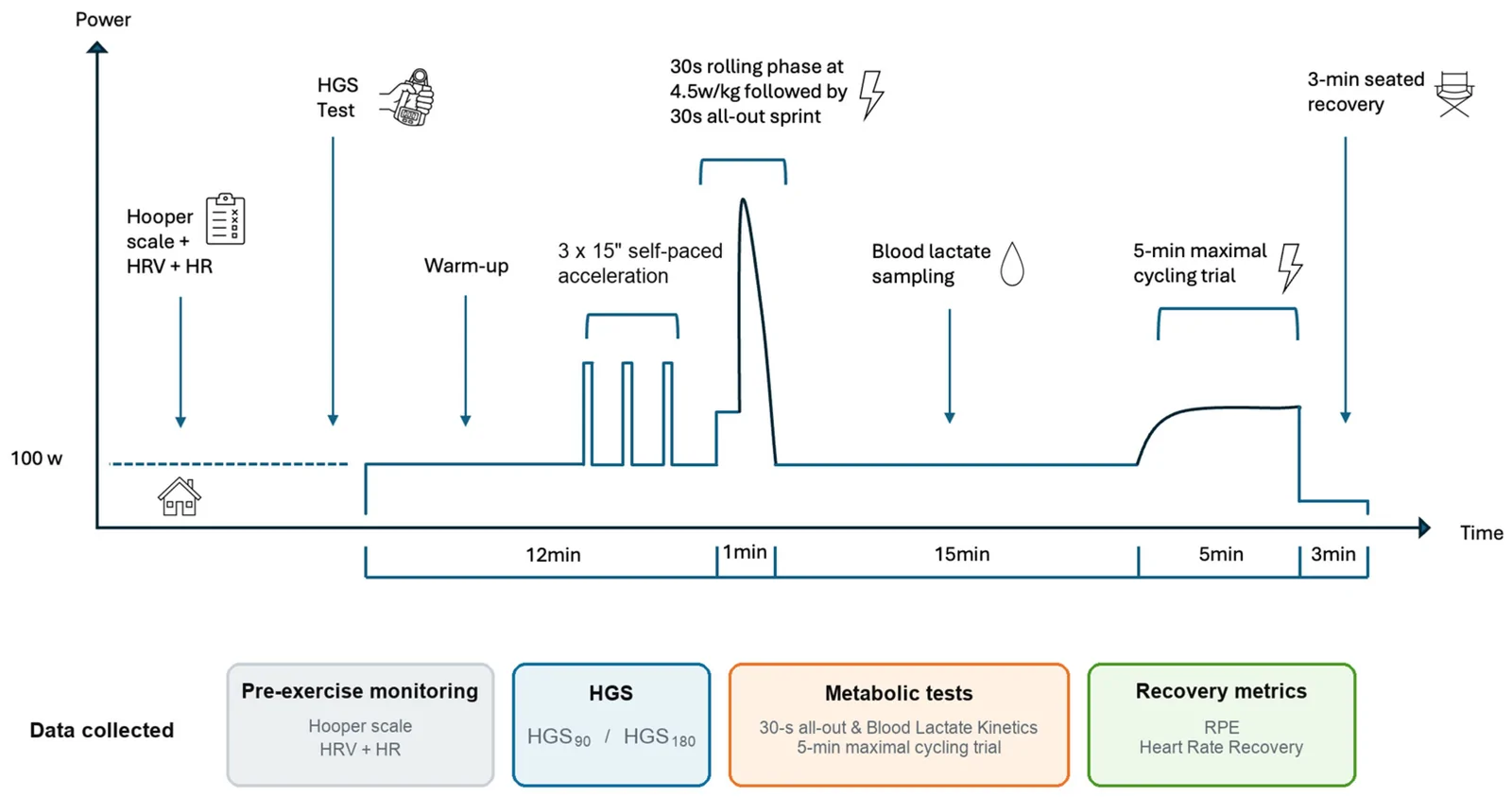
Schematic representation of the laboratory testing-day session. HRV = heart rate variability; HR = heart rate; HGS = handgrip strength test; RPE = rate of perceived exertion.

**Figure 3:**
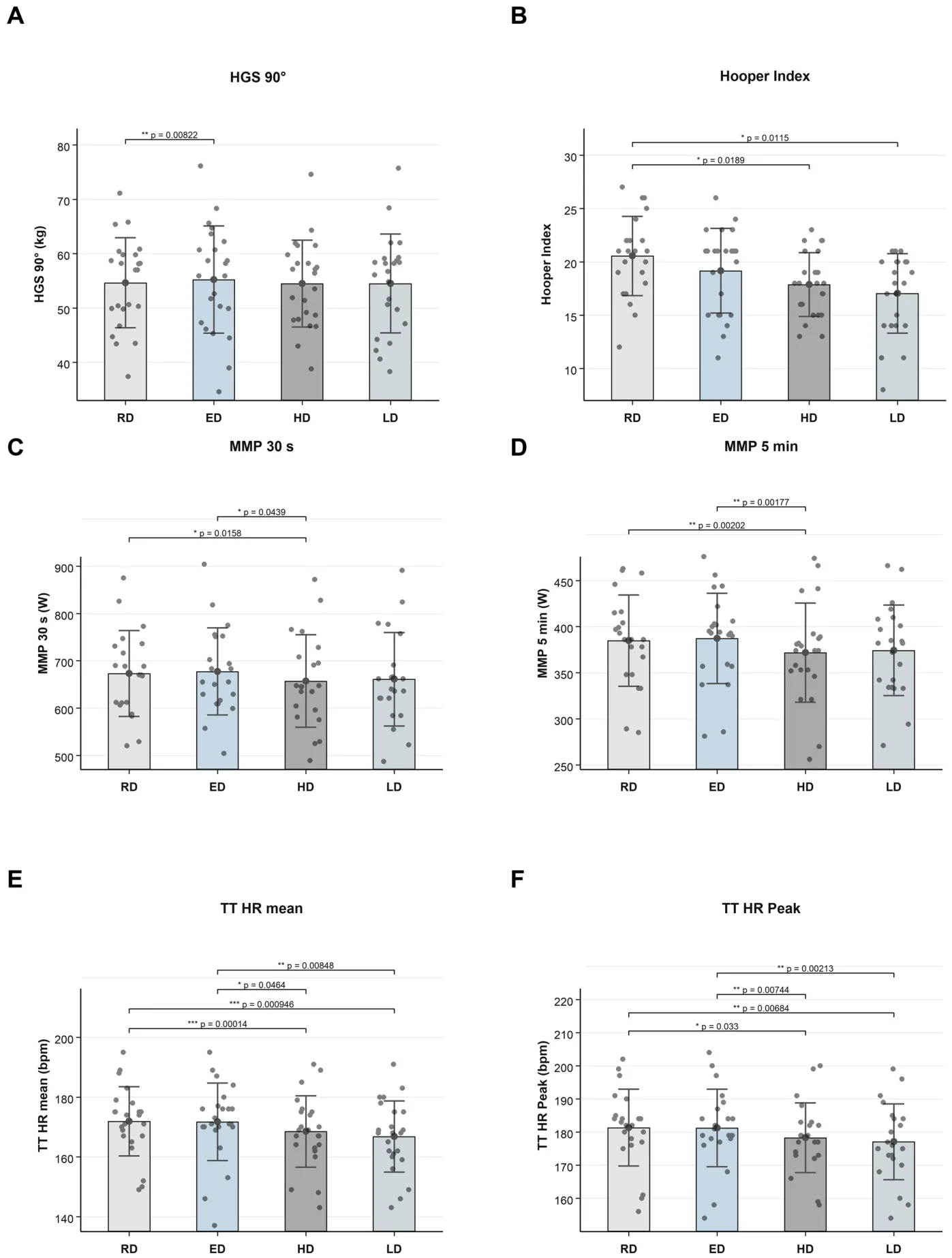
Selected repeated-measures outcomes across RD, ED, HD, and LD: (**A**) HGS_90_, (**B**) Hooper Index, (**C**) MMP_30s_, (**D**) MMP_5min_, (**E**) TT HRmean, (**F**) TT HRpeak. Statistical significance thresholds were set at *p* < 0.05 (*), *p* < 0.01 (**), *p* < 0.001 (***). Complete results for all outcomes are provided in Table 2.

**Figure 4:**
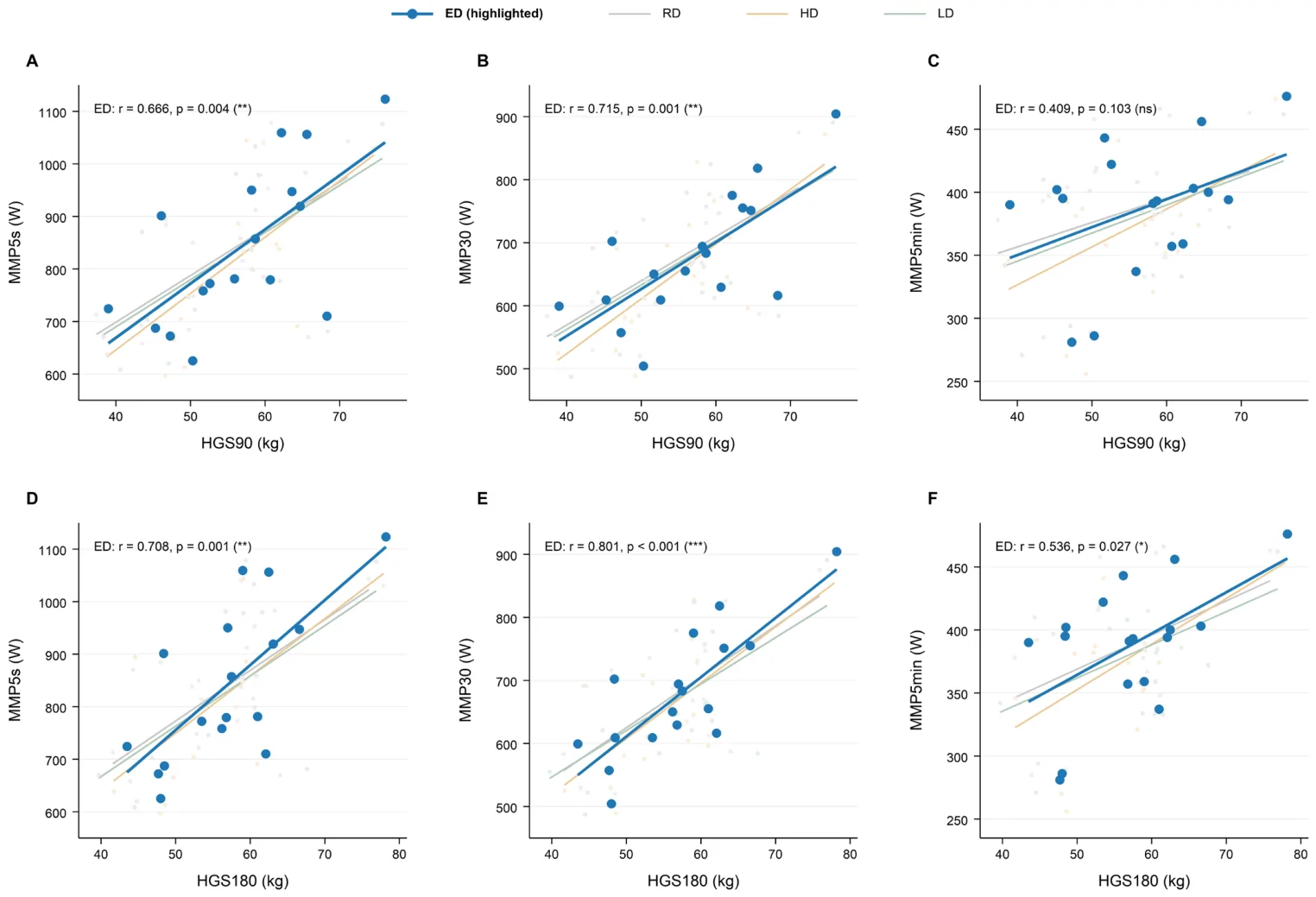
Condition-specific associations of handgrip strength with mean maximal power. Associations between (**A**) HGS_90_ and MMP_5s_; (**B**) HGS_90_ and MMP_30s_; (**C**) HGS_90_ and MMP_5min_; (**D**) HGS_180_ and MMP_5s_; (**E**) HGS_180_ and MMP_30s_; and (**F**) HGS_180_ and MMP_5min_. ED observations and regression lines are highlighted in blue, whereas relationships for RD, HD, and LD are shown in attenuated colors. Pearson’s correlation coefficients (r) and corresponding *p*-values are reported for ED. * *p* < 0.05; ** *p* < 0.01; *** *p* < 0.001; ns, not significant. HGS_90_, handgrip strength measured at 90° of elbow flexion; HGS_180_, handgrip strength measured at 180° of elbow flexion; MMP_5s_, 5-s mean maximal power; MMP_30s_, 30-s mean maximal power; MMP_5min_, 5-min mean maximal power; RD, rest day; ED, easy day; HD, hard day; LD, long day.

**Table 1:** Summary of the four experimental conditions and common laboratory assessments.

Condition	2 Days Before Testing	Day Before Testing	Testing Day
RD (rest)	90 min easy ride	Complete rest day	Morning HRV/HR + HI; body mass; HGS; 30 s all-out test; lactate kinetics; 5 min all-out test; HR recovery; RPE
ED (easy)	90 min easy ride	Easy/active-recovery ride; Borg CR10 RPE < 3	
HD (high intensity)	90 min easy ride	5 × 5 min intervals at maximal average power	
LD (long duration)	90 min easy ride	Session at least as long as the participant’s usual weekly long ride	

**Table 2:** Descriptive statistics and repeated-measures condition effects. Values are mean ± SD. Partial η^2^ is the omnibus effect size (small ≈ 0.01, medium ≈ 0.06, large ≈ 0.14). Statistical significance thresholds were set at *p* < 0.05 (*), *p* < 0.01 (**), *p* < 0.001 (***).

Outcome	RD	ED	HD	LD	*p*	Partial η^2^
HGS_90_ (kg)	55.11 ± 9.0	56.84 ± 9.6	54.97 ± 8.4	54.51 ± 9.9	0.030 *	0.168
HGS_180_ (kg)	56.24 ± 8.4	57.04 ± 8.5	55.25 ± 8.1	55.98 ± 9.5	0.133	0.109
HI	20.41 ± 3.4	18.29 ± 4.2	17.59 ± 2.5	16.76 ± 3.2	<0.001 ***	0.303
HRVmorning	63.88 ± 11.0	63.65 ± 11.2	67.12 ± 12.6	63.94 ± 11.5	0.247	0.084
HRmorning (bpm)	53.41 ± 7.7	51.82 ± 8.5	53.59 ± 7.4	54.65 ± 7.1	0.459	0.052
MMP_5s_ (W)	832.76 ± 130.8	842.35 ± 148.9	806.29 ± 144.2	819.88 ± 147.7	0.033 *	0.165
MMP_30s_ (W)	675.18 ± 100.2	677.06 ± 100.1	653.76 ± 105.8	664.47 ± 110.0	0.005 **	0.235
PDI (%)	38.25 ± 8.5	38.66 ± 7.9	38.40 ± 8.2	38.46 ± 8.4	0.991	0.002
MMP_5min_ (W)	385.82 ± 52.0	387.35 ± 52.1	371.53 ± 57.6	377.59 ± 52.1	<0.001 ***	0.381
BLaPeak (mmol/L)	7.85 ± 1.9	8.25 ± 2.2	7.53 ± 1.6	7.57 ± 2.1	0.176	0.097
TTP-BLa	5.45 ± 1.6	4.97 ± 1.1	5.42 ± 1.3	5.21 ± 1.6	0.608	0.037
BLaClearance	0.46 ± 0.1	0.47 ± 0.1	0.49 ± 0.2	0.52 ± 0.2	0.764	0.024
RPE	7.38 ± 1.5	7.44 ± 1.5	7.31 ± 1.8	7.56 ± 2.0	0.875	0.015
TT HRpeak (bpm)	180.47 ± 12.9	180.71 ± 13.0	177.71 ± 11.6	177.12 ± 12.6	<0.001 ***	0.398
TT HRmean (bpm)	170.71 ± 13.2	170.41 ± 14.7	166.71 ± 12.9	166.06 ± 13.1	<0.001 ***	0.410
Cadence (rpm)	102.24 ± 4.7	101.41 ± 4.2	100.94 ± 5.5	100.53 ± 5.3	0.191	0.093
HRR1 (bpm)	42.35 ± 11.3	44.00 ± 12.2	45.82 ± 16.2	44.76 ± 12.8	0.298	0.073
HRR3 (bpm)	80.41 ± 9.8	80.65 ± 8.4	84.12 ± 9.3	80.94 ± 9.5	0.060	0.142

## Data Availability

The data are accessible upon request to the authors.

## Abbreviations

The following abbreviations are used in this manuscript:

BLaClearance	Blood lactate clearance
BLaPeak	Blood lactate peak
CMJ	Countermovement jump
ED	Easy day
HD	High-intensity day
HGS	Handgrip strength
HGS_90_	Handgrip strength with a 90° elbow flexion
HGS_180_	Handgrip strength with an overhead arm position
HI	Hooper Index
HIT	High-intensity training
HRmorning	Resting heart rate
HRPeak	Peak heart rate
HRR	Heart rate recovery
HRVmorning	Heart rate variability collected in the morning
LD	Long day
MAP	Maximal aerobic power
PDI	Power decrement index
RD	Rest day
RPE	Rating of perceived exertion
RPO	Recorded power output
SD	Standard deviation
TT HRmean	Mean time-trial heart rate
TT HRpeak	Peak time-trial heart rate
TT RPM	Time-trial cadence
TTP-BLa	Time to blood lactate peak
